# Supplementary material for: The Small RNA Universe of Capitella teleta
Source: Front Mol Biosci. 2022 Feb 25;9:802814. doi: 10.3389/fmolb.2022.802814 (PMC8915122; doi:10.3389/fmolb.2022.802814)
Supplement: Supplementary file 1 [file DataSheet1.ZIP › Supplement/confident/CAPTEscaffold_60_5417.pdf]

Provisional ID	:	CAPTEscaffold_60_5417
Score total	:	5788.6
Score for star read(s)	:	3.9
Score for read counts	:	5782.1
Score for mfe	:	1.6
Score for randfold	:	1.6
Score for cons. seed	:	-0.6
Total read count	:	11353
Mature read count	:	11336
Loop read count	:	0
Star read count	:	17

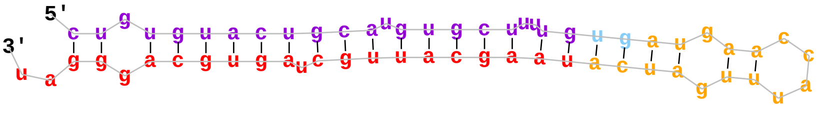

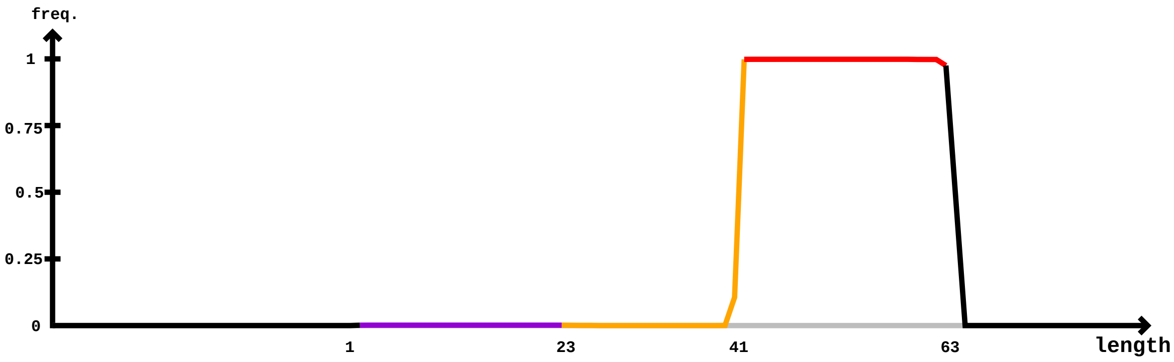

Star **Mature**

```
5'- ugaguuuccuauucuuuggcacucacucuucuguguacugcaugugcuuuugugaugaaccauuugaucauaagcauugcuagugcagggaugggugauguugguacagcua  -3'  obs
    ugaguuuccuauucuuuggcacucacucuucuguguacugcaugugcuuuugugaugaaccauuugaucauaagcauugcuagugcagggaugggugauguugguacagcua       exp
    .((((.....))))...((((.((((((.(((.(((((((((.(((((..((((((.((....)).))))))))))))).)))))).))).)))))))))))..........   reads   mm   sample
    ..............................cuguguacugcaugugcuuuu.............................................................   1       0    seq
    ..............................cuguguacugcaugugcuuuuA............................................................   1       1    seq
    ..............................cuguguacugcaugugcuuuug............................................................   5       0    seq
    ..............................cuguguacugcaugugcuuuugu...........................................................   4       0    seq
    ..............................cuguguacugcaugugcuuuuguga.........................................................   4       0    seq
    ...............................uguguacugcaugugcuuuuguga.........................................................   1       0    seq
    ...............................uguguacugcaugugcuuuugugG.........................................................   1       1    seq
    ....................................................................cauaagcauugcuagugcagggau....................   2       0    seq
    .....................................................................auaagcauugcuaguAcag........................   4       1    seq
    .....................................................................auaagcauugcuaguAcagg.......................   1       1    seq
    .....................................................................auaagcauugcuaguAcaggg......................   1       1    seq
    .....................................................................auaagcauugcuaguAcaggga.....................   30      1    seq
    .....................................................................auaagcauugcuagugcaggga.....................   1       0    seq
    .....................................................................auaagcauugcuagugcagggau....................   85      0    seq
    .....................................................................auaagcauugcuaguAcagggau....................   1062    1    seq
    .....................................................................auaagcauugcuagugcagggauU...................   3       1    seq
    .....................................................................auaagcauugcuaguAcagggaug...................   12      1    seq
    .....................................................................auaagcauugcuagugcagggauA...................   1       1    seq
    ......................................................................uaagcauugcuaguAcag........................   3       1    seq
    ......................................................................uaagcauugcuagugcaggga.....................   1       0    seq
    ......................................................................uaagcauugcuaguAcaggga.....................   226     1    seq
    ......................................................................uGagcauugcuagugcagggau....................   1       1    seq
    ......................................................................uaagcauugcuagugGagggau....................   1       1    seq
    ......................................................................uaagcauugcuagugcGgggau....................   1       1    seq
    ......................................................................uaagcauugcuaguAcagggau....................   8824    1    seq
    ......................................................................uaagcauugcuagugcagAgau....................   1       1    seq
    ......................................................................uaagcauuUcuagugcagggau....................   1       1    seq
    ......................................................................uaagAauugcuagugcagggau....................   1       1    seq
    ......................................................................uaagcauugcAagugcagggau....................   1       1    seq
    ......................................................................uaagcauugcuagugcagggau....................   407     0    seq
    ......................................................................uaagcauugcuaguAcagggaug...................   646     1    seq
    ......................................................................uaagcauugcuagugcagggauA...................   13      1    seq
    ......................................................................uaagcauugcuagugcagggauU...................   2       1    seq
```

```
                              Star                                    Mature
ugaguuuccuauucuuuggcacucacucuucuguguacugcaugugcuuuugugaugaaccauuugaucauaagcauugcuagugcagggaugggugauguugguacagcua
......................................................................uaagcauugcuagugcagggauC...................   1   1   seq
.......................................................................Uagcauugcuagugcagggau....................   1   1   seq
.......................................................................aagcauugcuaguAcagggau....................   2   1   seq
..........................................................................cauugcuaguAcagggau....................   1   1   seq
```
